# Supplementary material for: Identification of photoperiod-induced specific miRNAs in the adrenal glands of Sunite sheep (Ovis aries)
Source: Front Vet Sci. 2022 Jul 22;9:888207. doi: 10.3389/fvets.2022.888207 (PMC9354845; doi:10.3389/fvets.2022.888207)
Supplement: Supplementary file 1 [file Data_Sheet_1.ZIP › Supplementary file/miRDeep2 file.zip!/miRDeep2 file/Know_Structure/SP42A2/oar-mir-154a.pdf]

miRBase precursor : oar-mir-154a
Total read count : 856
oar-miR-154a-5p read count : 3
oar-miR-154a-3p read count : 848
remaining reads : 5

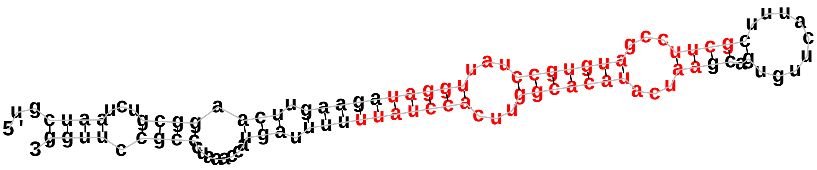

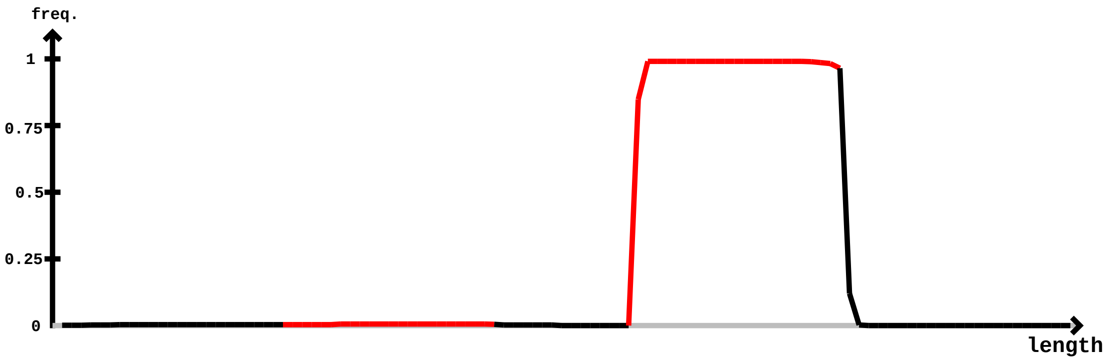

oar-miR-154a-5p

oar-miR-154a-3p

```
5'- ugcuaaucugcggaacuugaagauagguuauccguguagccuucgcuuuacuugugacgaaucauacacgguucaccuauuuuuuaguaccaaaucccgccuugg   -3'    exp
    .((......))((.(((.((((((((((...(((((((...(((((.........).))))...)))))))...)))))))))).))).))..............    reads   mm   sample
    ugcuaaucugcggaacuugaaga..................................................................................    1       0    seq
    ..cuaaucugcggaacuugaaga..................................................................................    1       0    seq
    .....aucugcggaacuugaaga..................................................................................    1       0    seq
    .......................uagguuauccguguagccuuc.............................................................    1       0    seq
    .......................uagguuauccguguagccuucg............................................................    2       0    seq
    ............................uauccguguagccuucgcuuuac......................................................    2       0    seq
    ...........................................................aaucauacacgguucacc............................    1       0    seq
    ...........................................................aaucauacacgguucaccu...........................    3       0    seq
    ...........................................................aaucauacacgguucaccua..........................    3       0    seq
    ...........................................................aaucauacacgguucaccuau.........................    15      0    seq
    ...........................................................aaucauacacgguucaccuauu........................    692     0    seq
    ...........................................................aaucauacacgguucaccuauuu.......................    12      0    seq
    ............................................................aucauacacgguucaccuauu........................    31      0    seq
    ............................................................aucauacacgguucaccuauuu.......................    89      0    seq
    ............................................................aucauacacgguucaccuauuuu......................    2       0    seq
```
